# Detection of Double-Stranded RNA Intermediates During SARS-CoV-2 Infections of Syrian Golden Hamsters with Monoclonal Antibodies and Its Implications for Histopathological Evaluation of In Vivo Studies

**DOI:** 10.3390/ijms252111425

**Published:** 2024-10-24

**Authors:** Georg Beythien, Madeleine de le Roi, Stephanie Stanelle-Bertram, Federico Armando, Laura Heydemann, Malgorzata Rosiak, Svenja Becker, Mart M. Lamers, Franziska K. Kaiser, Bart L. Haagmans, Malgorzata Ciurkiewicz, Gülşah Gabriel, Albert D. M. E. Osterhaus, Wolfgang Baumgärtner

**Affiliations:** 1 Department of Pathology, University of Veterinary Medicine Hannover, Foundation, 30559 Hannover, Germany; georg.beythien@tiho-hannover.de (G.B.); madeleine.de.le.roi@tiho-hannover.de (M.d.l.R.); federico.armando@unipr.it (F.A.); laura.heydemann@tiho-hannover.de (L.H.); malgorzata.rosiak@tiho-hannover.de (M.R.); svenja.becker@tiho-hannover.de (S.B.);; 2 Center for Systems Neuroscience, University of Veterinary Medicine Hannover, Foundation, 30559 Hannover, Germany; 3 Leibniz Institute of Virology, 20251 Hamburg, Germany; stephanie.stanelle-bertram@leibniz-liv.de (S.S.-B.); guelsah.gabriel@leibniz-liv.de (G.G.); 4 Pathology Unit, Department of Veterinary Science, University of Parma, 43121 Parma, Italy; 5 Department of Viroscience, Erasmus University Medical Center, 3000 CA Rotterdam, The Netherlands; mart@duke-nus.edu.sg (M.M.L.); b.haagmans@erasmusmc.nl (B.L.H.); 6 Emerging Infectious Diseases, Duke-NUS Medical School, Singapore 169857, Singapore; 7 Research Center for Emerging Infections and Zoonoses, University of Veterinary Medicine Hannover, Foundation, 30559 Hannover, Germany; franziska.kaiser@nih.gov (F.K.K.); albert.osterhaus@tiho-hannover.de (A.D.M.E.O.); 8 Institute of Virology, University of Veterinary Medicine Hannover, Foundation, 30559 Hannover, Germany

**Keywords:** SARS-CoV-2, Syrian golden hamsters, viral replication, variants of concern, dsRNA

## Abstract

The SARS-CoV-2 pandemic has highlighted the challenges posed by the emergence and rapid global spread of previously unknown viruses. Early investigations on the pathogenesis of newly identified viruses are often hampered by a lack of appropriate sample material and conventional detection methods. In this study, viral replication within the lungs of SARS-CoV-2-infected Syrian golden hamsters was assessed by immunolabeling dsRNA intermediates with three different monoclonal antibodies in formalin-fixed, paraffin-embedded tissue samples. The presence of dsRNA was compared to viral antigen levels, viral titers, and genomic RNA replicates using three different variants of concern and an ancestral virus strain at a single time point and during the course of infection with an ancestral variant, and then validated using fluorescent 2-plex in situ hybridization. The results indicate that the detection of viral infection using anti-dsRNA antibodies is restricted to an early phase of infection with high viral replication activity. Additionally, the combined detection of dsRNA intermediates and viral antigens may help to bridge the interpretation gaps between viral antigen levels and viral titers at a single time point. Further testing in other viral infections or species is needed to assess the potential of dsRNA as an early marker for viral infections.

## 1. Introduction

As the severe acute respiratory syndrome coronavirus 2 (SARS-CoV-2) pandemic has recently shown, science and society face major challenges when previously unknown viruses suddenly emerge and spread worldwide within a short period. The first SARS-CoV-2 case was registered in China in December 2019 and the disease was declared a pandemic within only 4 months [1,2,3,4,5]. Once a new emerging virus has been identified, the most important challenge is the establishment of robust virus identification methods and intervention strategies to combat the propagation of the virus. However, this requires a detailed and rapid molecular characterization of previously unknown agents. The first studies investigating the pathogenesis of SARS-CoV-2 were restricted by an uncertainty with regard to suitable animal models and limited access to organ samples from infected individuals [6,7,8,9]. In addition, analyses could often be performed only on virus-infected tissue from a single time point and without knowledge about the exact time of infection [6,7,10].

Investigations on cell tropism as well as the temporo-spatial distribution of viral antigens and replication sites within a host are crucial for understanding underlying pathological mechanisms [6]. Immunohistochemical detection and characterization of viral antigens using commercially available, virus-specific antibodies is a conventional method often performed in pathological investigations and virus characterization [6,11,12,13,14]. Although this is an inexpensive and time-efficient method for investigating virus presence and distribution, the targeted proteins have to be identified first. The production of commercially available antibodies requires a certain period of time after the initial identification of a new virus, which restricts their usability in virus discovery [2,15,16,17]. Additionally, it should be considered that while immunopositive reactions indicate the presence of viral components, they neither necessarily represent infectious viral particles nor do they allow for conclusions to be made concerning the activity of the infection, and may only represent residual fragments of viruses [6]. Parts of these limitations can be overcome by the employment of additional molecular techniques, including polymerase chain reaction (PCR) and in situ hybridization (ISH), for the detection of viral genomes and the in vitro detection of infectious viral particles [18,19,20,21]. However, using and interpreting these techniques is also dependent on an initial sequencing of the viral genome as well as a relatively precise knowledge of viral replication behavior and the availability of suitable cell lines. A potential solution to this challenge is the antibody-based detection of intermediate products of viral replication.

**Figure 1:**
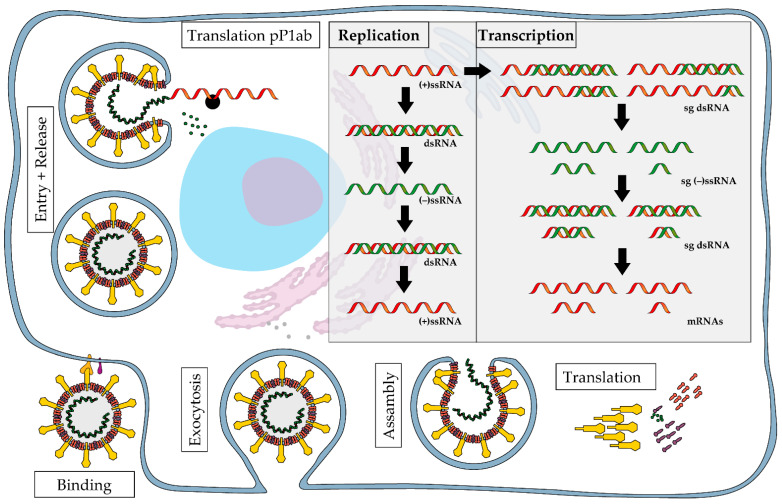
Schematic illustration of SARS-CoV-2 replication cycle and formation of double-stranded RNA intermediates. (+)ssRNA, positive-sense single-stranded RNA; (−)ssRNA, negative-sense single-stranded RNA; dsRNA, double-stranded RNA; sg, sub-genomic; pP1ab, protein replicase polyprotein 1ab.

During viral replication and/or translation, viruses are forced to produce reverse-oriented copies of their genome or messenger RNAs to effectively utilize the host cell machinery and generate viral progeny. During that process, certain amounts of double-stranded RNA (dsRNA) intermediates are formed by the complementary binding of negative- and positive-sense RNA molecules, as illustrated in Figure 1 for the replication cycle of SARS-CoV-2. DsRNA is therefore considered to provide evidence of ongoing viral replication in most RNA and DNA viruses [6,7,11,12,13,22,23,24]. Especially in single-stranded RNA (ssRNA) viruses, both the detection of anti-genomic RNA and the presence of dsRNA intermediates are considered indicative of viral replication [6,24,25,26]. Therefore, antibodies sensing dsRNA can be employed both as a pan-viral detection tool for viral infections in general and for the demonstration of viral replication in particular [7,22,25,27,28]. This indicates that anti-dsRNA antibodies provide a versatile alternative method for investigating viral pathogenesis.

In addition, the presence of a pathogen alone does not provide evidence of whether the pathogen is the causative agent for a disease or represents a commensal or contamination [29]. To overcome difficulties such as virus isolation and virus cultivation in order to establish a causal relationship between the newly emerging virus and the observed disease and thus fulfill Koch’s postulates, the modified Koch’s postulates can alternatively be used to demonstrate intralesional viral nucleic acids [29,30,31,32,33,34].

The aim of this study was to visualize viral replication through immunofluorescent labeling of dsRNA intermediates with three different commercially available antibodies in comparison to the amount of viral antigen present in formalin-fixed, paraffin-embedded (FFPE) lung specimens of Syrian golden hamsters (SGHs; *Mesocricetus auratus*) experimentally infected with SARS-CoV-2. In the first experiment, hereafter referred to as experiment 1, dsRNA expression was investigated in animals infected with the ancestral strain 614G_Bavpat-1_ and the variants of concern (VOCs) Gamma, Delta, and Omicron at 4 days post infection (dpi). In the second experiment, hereafter referred to as experiment 2, dsRNA expression was investigated over the course of an infection with the ancestral strain 614G_Hamburg_ at 1, 3, and 6 dpi. The presence of dsRNA intermediates in both experiments was correlated with detected levels of genomic RNA as well as infectious viral titers at 4 dpi and during the course of the infection. DsRNA expression was verified by fluorescent in situ hybridization (FISH).

## 2. Results

### 2.1. Comparison of SARS-CoV-2 S and SARS-CoV-2 NP Expression in SARS-CoV-2-Infected Syrian Golden Hamsters

Within experiment 1, viral antigen was observed in all infected animals. Immunopositive signals for SARS-CoV-2 S and SARS-CoV-2 NP were detected cytoplasmically in bronchial epithelial cells and within the alveolar compartment of lungs from hamsters infected with 614G_Bavpat-1_ and with the VOCs Gamma, Delta, and Omicron. The quantitative analysis of SARS-CoV-2-positive cells revealed considerable differences between the VOCs. The percentage of SARS-CoV-2-immunopositive cells was significantly higher in Gamma-infected hamsters compared to hamsters infected with the Omicron variant. However, on average, more than 50% of the cells expressed SARS-CoV-2 S in all four virus variants (Supplementary Figure S1A).

For experiment 2, the highest percentage of SARS-CoV-2-immunopositive cells was identified at 3 dpi. Lower percentages of virus antigen-labeled cells were found at 1 dpi and only individual cells could be observed at 6 dpi. No specific immunoreactivity for SARS-CoV-2-specific antigens was observed in the mock-infected SGHs. The vast majority of labeled cells expressed the SARS-CoV-2 S antigen. In contrast, only lower proportions of SARS-CoV-2 NP were detected (Supplementary Figure S1B).

As SARS-CoV-2 S expression was considerably higher compared to NP expression, the total number of SARS-CoV-2 S-labeled cells within this assay was used as a reference for further analysis.

### 2.2. Detection of dsRNA in SARS-CoV-2-Infected Syrian Golden Hamsters

**Figure 2:**
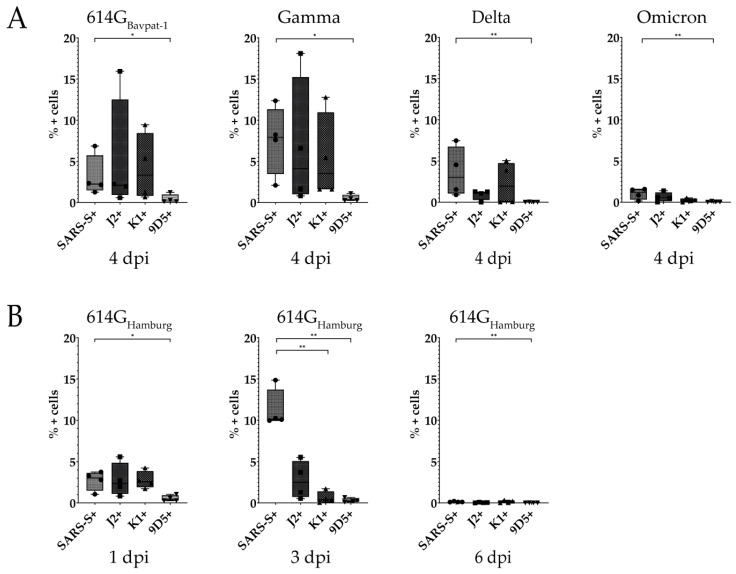
Comparison of SARS-CoV-2 spike protein (S) and double-stranded RNA (dsRNA) detection with monoclonal anti-dsRNA antibodies J2, K1, and 9D5 in SARS-CoV-2-infected hamsters. (**A**) Detection of SARS-CoV-2 S and dsRNA at 4 days post infection (dpi) in animals infected with 614G_Bavpat-1_ and with the variants of concern (VOCs) Gamma, Delta, and Omicron. (**B**) SARS-CoV-2 S and dsRNA detection at 1, 3, and 6 dpi with the early strain 614G_Hamburg_. Data are shown as box and whisker plots. The boundaries of the box plot indicate the 25th and 75th percentiles, the bar indicates the median, and the whiskers indicate the minimum and maximum. Data were analyzed by two-tailed Mann–Whitney U tests, followed by the Benjamini–Hochberg correction. A *p*-value of ≤0.05 was chosen as the cut-off for statistical significance. N = 4 animals/group. Exact *p*-values are represented as *, with * *p* < 0.05 and ** *p* < 0.01.

DsRNA formation was demonstrated in the cytoplasm of the bronchial epithelial cells and alveoli of SARS-CoV-2-infected hamsters by immunofluorescent labeling with three different anti-dsRNA antibodies. In experiment 1, all three anti-dsRNA antibodies yielded immunopositive reactions for dsRNA in all virus variants. Although the results showed corresponding changes to viral antigen detection in terms of the amount of immunolabeled cells, varying detection levels between individual anti-dsRNA antibodies were observed (Figure 2). While in hamsters infected with 614G_Bavpat-1_ and Gamma, a considerable expression of dsRNA could be detected, only low numbers of dsRNA immunopositive cells could be identified in Delta- and Omicron-infected hamsters. The application of the J2 antibody yielded high detection levels in 614G_Bavpat-1_-, Gamma-, and Omicron-infected animals. Similar amounts of dsRNA expression with less variation could be observed for K1 labeling in SARS-CoV-2 614G_Bavpat-1_- and Gamma-infected animals. Interestingly, within Delta-infected animals, two individuals showed a considerably higher amount of dsRNA with K1 compared to detection with J2, while detection in two other animals was considerably lower. A closer investigation of K1 dsRNA immunolabeling in those two animals revealed immunopositive signals in close proximity to the foci of viral antigen staining that did not correspond to the FISH stainings of SARS-CoV-2-specific RNAs (Supplementary Figures S2 and S3). The labeling of dsRNA with 9D5 resulted in significantly lower amounts of immunopositive cells compared to detection with J2, K1, and SARS-CoV-2 S protein (Figure 2A).

In experiment 2, similar detection levels of dsRNA expression and viral antigens were observed at 1 dpi, while dsRNA detection was considerably reduced at 3 dpi when viral antigen detection was the highest. At 6 dpi, viral antigen as well as dsRNA detection were close to zero. As already observed for experiment 1, detection levels varied between anti-dsRNA-specific antibodies. The highest individual amounts of dsRNA-positive cells and broadest variation were noticed for J2 at all time points, followed by the detection with K1. As in experiment 1, 9D5 immunolabeling resulted in a significantly reduced number of immunopositive cells compared to SARS-CoV-2 antigen detection (Figure 2B). In mock-infected control hamsters, the investigation for the presence of dsRNA resulted only in non-specific immunopositive reactions.

### 2.3. Co-Detection of dsRNA and SARS-CoV-2 S in SARS-CoV-2 614G_Bavpat-1_ and VOCs

**Figure 3:**
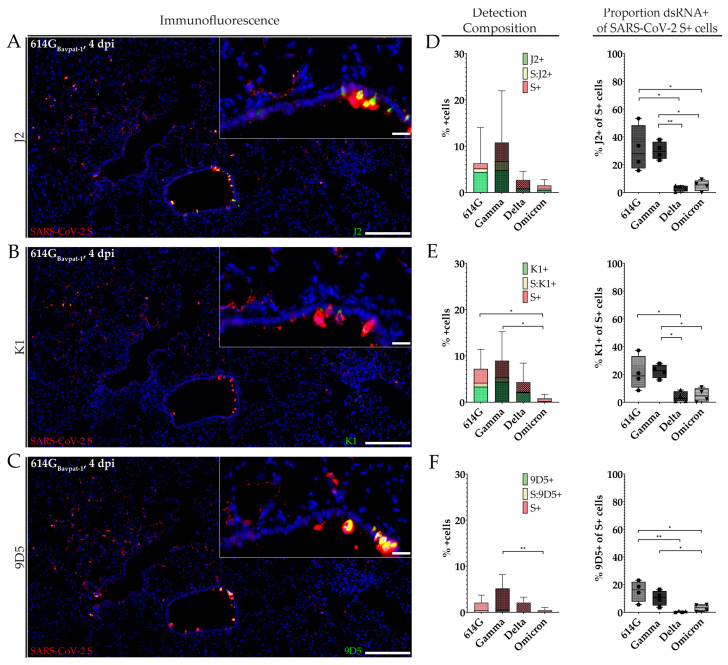
Comparative assessment of double-stranded RNA (dsRNA) expression and SARS-CoV-2-immunopositive cell levels with three anti-dsRNA antibodies in hamsters infected with SARS-CoV-2 614G_Bavpat-1_ and the variants of concern (VOCs) Gamma, Delta, and Omicron at 4 days post infection (dpi). (**A**–**C**): Representative images of dsRNA and SARS-CoV-2 spike (S) co-detection in an animal infected with 614G_Bavpat-1_ at 4 dpi. (**D**–**F**): Mean composition of cell detection and proportion of dsRNA-expressing cells within the total amount of SARS-CoV-2 S-labeled cells for J2 (**D**), K1 (**E**), and 9D5 (**F**). Bars indicate 200 µm and 20 µm (insert), respectively (**A**–**C**). Data are shown as box and whisker plots (**D**–**F**). The boundaries of the box plot indicate the 25th and 75th percentiles, the bar indicates the median, and the whiskers indicate the minimum and maximum. Data were analyzed by two-tailed Mann–Whitney U tests, followed by the Benjamini–Hochberg correction. A *p*-value of ≤0.05 was chosen as the cut-off for statistical significance. N = 4 animals/group. Exact *p*-values are represented as *, with * *p* < 0.05 and ** *p* < 0.01.

Closer analysis of SARS-CoV-2 S and dsRNA co-expression within the lungs of animals infected with SARS-CoV-2 614G_Bavpat-1_ and the VOCs Gamma, Delta, and Omicron revealed varying compositions of immunolabeled cell fractions between anti-dsRNA antibodies as well as varying proportions of dsRNA co-labeled cells between virus variants (Figure 3).

For all anti-dsRNA antibodies, co-localization with SARS-CoV-2 S labeling was observed within the bronchial epithelia and alveolar septa (Figure 3A–C and Figure S3) and corresponded with 2-plex FISH (Supplementary Figure S2A). An investigation of the detection composition revealed considerable amounts of cells exclusively labeled for dsRNA and varying amounts of cells co-expressing viral antigen and dsRNA in 614G_Bavpat-1_- and Gamma-infected animals. In contrast, the majority of immunopositive cells in the VOCs Delta and Omicron were composed of cells exclusively expressing viral antigen. Additionally, only very low amounts of cells co-expressing dsRNA and viral antigen were observed for those variants (Figure 3D–F). Investigation of the SARS-CoV-2 S-positive cell population revealed strain-specific differences in dsRNA expression at 4 dpi. While significantly higher amounts of dsRNA co-labeled cells were found in 614G_Bavpat-1_- and Gamma-infected animals, only very low levels of co-expressing cells were observed in Delta- and Omicron-infected animals. This is especially noteworthy, as no differences in viral antigen expression were seen between 614G_Bavpat-1_-, Gamma-, and Delta-infected animals (Supplementary Figure S1).

### 2.4. Co-Detection of dsRNA and SARS-CoV-2 S During Infection with SARS-CoV-2 614G_Hamburg_

**Figure 4:**
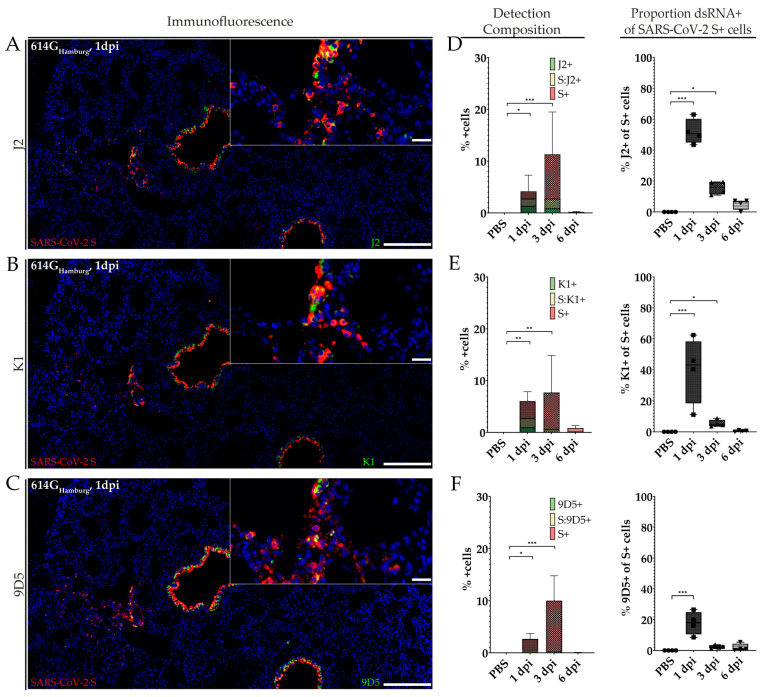
Comparative assessment of double-stranded RNA (dsRNA) and SARS-CoV-2 immunopositive cell levels with three anti-dsRNA antibodies in hamsters infected with SARS-CoV-2 614G_Hamburg_ at 1, 3, and 6 days post-infection (dpi) and mock-infected control animals (phosphate-buffered saline, PBS). (**A**–**C**): Representative images of dsRNA and SARS-CoV-2 spike protein (S) co-detection in animals infected with 614G_Hamburg_ at 1 dpi. (**D**–**F**): Mean composition of cell detection and proportion of dsRNA-expressing cells within the total amount of SARS-CoV-2 S-labeled cells for J2 (**D**), K1 (**E**), and 9D5 (**F**). Bars indicate 200 µm and 20 µm (insert), respectively (**A**–**C**). Data are shown as box and whisker plots (**D**–**F**). The boundaries of the box plot indicate the 25th and 75th percentiles, the bar indicates the median, and the whiskers indicate the minimum and maximum. Data were analyzed by two-tailed Mann–Whitney U tests, followed by the Benjamini–Hochberg correction. A *p*-value of ≤0.05 was chosen as the cut-off for statistical significance. N = 4 animals/group. Exact *p*-values are represented as *, with * *p* < 0.05, ** *p* < 0.01, and *** *p* < 0.001.

Immunopositive cells expressing SARS-CoV-2 S and dsRNA were detected in SARS-CoV-2 614G_Hamburg_-infected SGHs at all three time points of infection. One day post infection, mainly bronchial epithelial cells tested positive for both viral antigen and dsRNA. At 3 dpi, viral antigen and dsRNA were more often observed in alveoli. In contrast, at 6 dpi, only single cells tested positive for viral antigen (Figure 4A–C). Using FISH, genomic and anti-genomic RNA could be detected in corresponding areas (Supplementary Figure S2). Analysis of the amount of immunopositive cells revealed a substantially increasing number from 1 dpi to 3 dpi. In contrast, immunopositive cells could scarcely be observed at 6 dpi. The composition of the detected immunopositive cells corresponded to experiment 1. At 1 dpi, up to 50% of the immunopositive cells expressed dsRNA, while the percentage of cells expressing dsRNA had already declined considerably at 3 dpi. At 6 dpi, only the small foci of cells expressed viral antigen or dsRNA and no co-localization was observed. A closer analysis of SARS-CoV-2 S-immunopositive cells showed a steady decline in the number of double-labeled cells over the course of infection (Figure 4D–F and Figure S4). As observed for experiment 1, dsRNA detection with K1 was present around the foci of viral antigen and did not correspond to SARS-CoV-2-specific FISH signals (Supplementary Figure S2).

### 2.5. Correlation of dsRNA Expression with Viral Antigen, mRNA Levels, and Viral Titers

**Figure 5:**
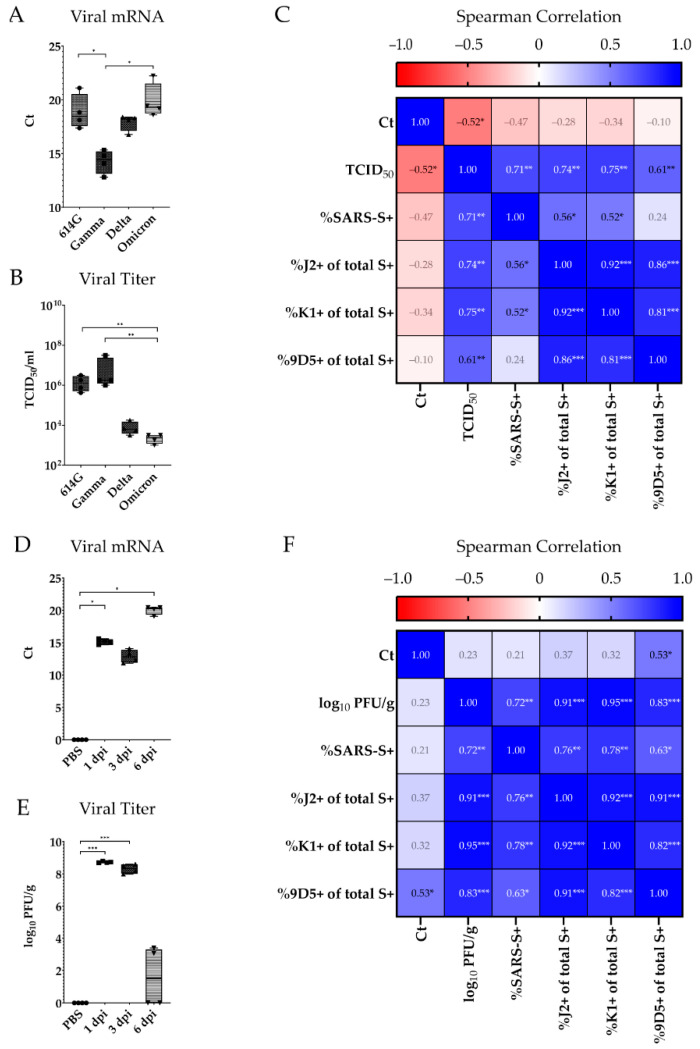
Virological assessment and correlation with immunolabeling data. (**A**): Viral mRNA detection by quantitative real-time polymerase chain reaction (qRT-PCR) in 614G_Bavpat-1_- and variant of concern (VOC)-infected animals at 4 days post infection (dpi). (**B**): Infectious viral titers assessed by TCID_50_ assays. (**C**): Spearman correlation of viral mRNA detection, viral titers, viral antigen detection, and double-stranded RNA (dsRNA) expression within SARS-CoV-2-positive stained cells. (**D**): Viral mRNA detection by qRT-PCR in 614G_Hamburg_-infected animals and mock-infected controls at 1, 3, and 6 dpi. (**E**): Infectious viral titers assessed as plaque-forming units (PFUs). (**F**): Spearman correlation of viral mRNA detection, viral titers, viral antigen detection, and dsRNA expression within SARS-CoV-2-positive stained cells. mRNA levels as well as viral titers were published previously and were extracted from the original datasets [35,36]. Data are shown as box and whisker plots. The boundaries of the box plot indicate the 25th and 75th percentiles, the bar indicates the median, and the whiskers indicate the minimum and maximum. Data were analyzed by two-tailed Mann–Whitney U tests, followed by the Benjamini–Hochberg correction (**A**,**B**,**D**,**E**). Correlations were calculated as nonparametric Spearman r values (**C**,**F**). A *p*-value of ≤0.05 was chosen as the cut-off for statistical significance. N = 4 animals/group. Exact *p*-values are represented as *, with * *p* < 0.05, ** *p* < 0.01, and *** *p* < 0.001.

For a more detailed investigation of dsRNA expression in comparison to other virological assay data and findings, viral mRNA levels and viral titers were extracted from the original study datasets published previously [35,36]. For experiment 1, the highest cycle threshold (Ct) values were observed within the lungs of 614G_Bavpat-1_- and Omicron-infected animals. The lowest Cts were seen in Gamma-infected animals, followed by intermediate values for Delta-infected animals (Figure 5A) [35]. Infectious viral titers were highest within 614G_Bavpat-1_- and Gamma-infected animals and low within Delta- and Omicron-infected animals (Figure 5B). Concerning the correlation of viral titers with the proportion of SARS-CoV-2 S-positive cells co-expressing dsRNA, high correlation values were observed for all three antibodies (Figure 5C) [35]. Within experiment 2, Ct values were highest at 6 dpi, moderate at 1 dpi, and lowest at 3 dpi (Figure 5D) [36]. Viral titers declined from 1 dpi to 6 dpi, as shown previously (Figure 5E) [36]. The correlation with the proportion of SARS-CoV-2 S-positive cells co-expressing dsRNA revealed an insignificant correlation with viral mRNA detection but a strong positive correlation with viral titers and dsRNA, as seen for experiment 1 (Figure 5F).

## 3. Discussion

### 3.1. DsRNA-Specific Antibodies as an Alternative Detection System for SARS-CoV-2 and Implications for Other Viral Agents with Consideration of Detection Timeframes for Different Methods

The first aim of this study was to examine the usability of three commercially available anti-dsRNA antibodies as alternative virus detection markers for SARS-CoV-2 infections. In both experimental approaches, positive immunolabeling could be observed with all three tested antibodies as reported in previous studies [7]. However, the results differed substantially between variants, time points, and antibodies. Overall, J2 labeling performed best for the detection of SARS-CoV-2 infections, while 9D5 showed the lowest detection efficacy and significant differences in viral antigen detection. To a certain extent, this is consistent with the observations of a study by Lean et al. (2020), in which the investigation of dsRNA using J2 led to similar immunostaining, but 9D5 remained negative in FFPE SARS-CoV-2-infected cells [7]. While the level of detection was similar to viral antigen expression for 614G_Bavpat-1_- and Gamma-infected animals in experiment 1, an unexpected drop in dsRNA expression was observed for Delta-infected animals, suggesting deviations in viral replication activity between the variants. This became more evident when investigating dsRNA expression during the course of 614G_Hamburg_ infection in experiment 2. The infected animals showed similar amounts of dsRNA expression and viral antigen at 1 dpi, while this proportion changed to high amounts of viral antigen with lower expression of dsRNA at 3 dpi. At 6 dpi, dsRNA detection and viral antigen expression were close to zero, as expected for SARS-CoV-2 infections in SGHs characterized by an abrupt elimination of virus after the acute phase of infection [37]. The alternative detection of SARS-CoV-2 infections by applying anti-dsRNA antibodies therefore highly depends on viral replication activity and only shows comparable results in combination with viral antigen detection in the early phase of SARS-CoV-2 infections. Interesting results were additionally observed for K1 staining. Especially within the lungs of animals infected with the VOC Delta and in SGHs infected with 614G_Hamburg_ at 6 dpi, dsRNA expression around the foci of viral antigen was observed that did not correspond with the FISH detection of dsRNA intermediates. Even though J2 and K1 are considered to recognize dsRNA intermediates equal to or greater than 30 base pairs, the immunostaining suggests the detection of different dsRNA species [38]. Interestingly, these K1 interactions seem to be especially present at late stages of infection, when overall viral replication is low. The signal might therefore represent either remnants of viral intermediates or specific kinds of endogenous RNAs, including microRNA, small interfering RNA, the presence of dsRNA molecules due to defects in processing and degradation, and dsRNA molecules as a result of cellular stress reactions to previous viral infections of the tissue [39]. It is also assumed that endogenous dsRNA molecules can also activate the innate immune system via sensing by host cellular pattern recognition receptors (PAMPs) [40]. Since differentiation between endogenous dsRNA and viral dsRNA intermediates by PAMPs is apparently not possible, it cannot be ruled out that anti-dsRNA antibodies also recognize these cell-derived dsRNA molecules, even if their nucleotide length is usually below the detection level of the antibodies [39,40]. However, further in vitro assays and investigations of different viral agents are needed to substantiate this hypothesis. Although high dsRNA levels strongly indicate viral infections, their absence cannot exclude a potential viral infection. Considering this limitation, anti-dsRNA antibodies should not be used to rule out a viral infection, but can offer compelling evidence of viral replication in experimental settings.

### 3.2. Co-Detection of dsRNA and Viral Antigens to Assess Viral Replication Within Tissues

As the dsRNA detection results suggested variations in viral replication activity within experiments 1 and 2, the composition of the identified cell populations within SARS-CoV-2 S and dsRNA double labeling was investigated in more detail. Besides the expected fractions of double-labeled cells and cells exclusively expressing the SARS-CoV-2 S antigen, a third portion of cells solely expressing dsRNA was found in both experiments by using all three antibodies (Supplementary Table S1). This can be attributed to a combination of different effects. One possible explanation could be the presence of infected cells expressing dsRNA and with the simultaneous absence of sufficient translation of viral proteins for efficient immunolabeling. The automated detection of cells might represent another possible cause of immunolabeled cell detection. QuPath detects cells based on thresholds for nuclear counterstaining and antibody-specific stainings are detected within an expanded, hypothetical cell body around and within the detected nucleus without regard to actual cell borders. While this allows a sufficient approximation of lung tissue, the method is imprecise and the incorrect assignment of immunopositive signals cannot be avoided. Additionally, steric effects during co-staining cannot be ruled out completely [41]. However, viral replication activity could be evaluated best when the proportion of dsRNA-positive cells within SARS-CoV-2 S-positive cells was analyzed. Within experiment 1, high levels of viral replication could be observed in 614G_Bavpat-1_- and Gamma-infected animals, while only low replication activity was seen in Delta- and Omicron-infected animals. This was substantiated by the results of experiment 2, where viral replication activity was highest during early infection and declined with peaking viral antigen expression at 3 dpi and 6 dpi. The results of both experiments additionally correspond to the in vitro data of SARS-CoV-2 growth kinetics that show the prolonged infectivity of ancestral strains of SARS-CoV-2 as well as Gamma infections compared to the Delta and Omicron variants [42,43]. However, the VOCs Delta and Omicron have shown distinct differences in their transmission dynamics and pathogenicity in vivo [35,44,45,46,47]. While Omicron strains have been shown to replicate more abundantly in nasal epithelial cells and tracheal tissues compared to Delta strains, they showed reduced replication in pulmonary cells. This may, at least in part, explain their higher transmissibility and lower pathogenicity observed in Syrian golden hamsters [45,48,49]. These observations align with the presented data and with our earlier observations in experiment 1 [35]. However, in addition to the relevance of the compartment in which effective replication occurs, VOC transmission levels are influenced by a variety of other factors, including virus generation intervals, assembly and cell entry efficiency, and immune evasion [44,50,51,52]. In combination with the overall performance of detection, the simultaneous detection of dsRNA with J2 and viral antigen can therefore be used in samples with an unknown time point of infection to classify them into early or late infection phases. This can be of specific interest when the study design only allows for a single time point and the different infection kinetics need to be considered. The employment of anti-dsRNA antibodies therefore represents a useful auxiliary technique in the scientific toolbox for the evaluation of in vivo studies with restricted time points or the reduced availability of samples for virological investigation. The latter may also be an interesting aspect for retrospective analyses of archived specimens.

### 3.3. Implications of dsRNA Expression Levels in Combination with Virological Assay Data

To verify this assumption, the expression of viral antigen and dsRNA intermediates was correlated with viral RNA detection via qRT-PCR and titration results published previously [35,36]. When correlating the data at 4 dpi, proportions of double-labeled cells displayed a strong positive correlation with infectious viral titers for all three investigated anti-dsRNA antibodies. The data correlation in experiment 2 revealed an even stronger correlation of double-labeled cells with infectious viral titers. Even though the investigated methods have different sensitivities, targets, and differences in the coverage of processed tissue, these results indicate a strong association of dsRNA expression and infectious titers in SARS-CoV-2 infections. While all values aligned during the course of experiment 2, the assessment of viral dsRNA expression filled an explanatory gap between viral antigen expression and viral titers in Delta-infected animals in experiment 1. This underlines the applicability of anti-dsRNA antibody staining at a single time point of infection or retrospective analyses of archived FFPE specimen. However, as dsRNA is an intermediate step of viral replication, its detection depends highly on the replication strategy and amount of dsRNA formed during the replication of members of different virus families [22,25]. Consequently, investigations on the use of dsRNA antibodies indicate considerable species- and virus-specific differences in dsRNA expression patterns. The usability of these antibodies should therefore be assessed for a broader range of viruses before final statements are made about their general usability in this context.

## 4. Materials and Methods

### 4.1. Experimental Setup and Infection of Animals

**Figure 6:**
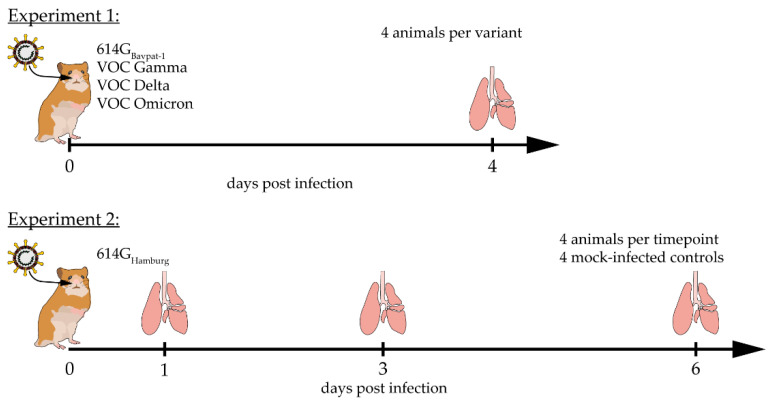
Study design. In experiment 1, four animals were intranasally infected with one of four different viruses, including 614G_Bavpat-1_ or the variants of concern (VOCs) Gamma, Delta, and Omicron, and euthanized at 4 days post infection (dpi). In experiment 2, Syrian golden hamsters were intranasally infected with the ancestral SARS-CoV-2 strain 614G_Hamburg_ and four animals were euthanized at 1, 3, and 6 dpi.

The FFPE lung samples included in this study were obtained from selected, experimentally infected 8-to-10-week-old male and female SGHs that had been used as untreated control groups in two previously published animal experiments [35,36,37,53,54,55,56]. Detailed information about the animals and viruses investigated in this study can be found in Supplementary Table S1. Briefly, animals were obtained from Janvier Laboratories (Le Genest-Saint-Isle, France) and kept under standardized environmental conditions. All infections were performed in biosafety level 3 laboratories in accordance with national and European animal welfare legislation. All infections were performed intranasally. The experimental setup of both experiments is illustrated in Figure 6.

In experiment 1, groups of four animals were infected with 10^4^ TCID_50_ of the ancestral strain SARS-CoV-2 614G_Bavpat-1_ or the VOCs Gamma, Delta, and Omicron as previously published [35,53]. At 4 dpi, the animals were euthanized and lung samples were collected and fixed in 10% neutral buffered formalin during subsequent necropsies as published previously [35]. The experimental infection of animals from experiment 2 was published by Stanelle-Bertram et al. (2023), Allnoch et al. (2021), Becker et al. (2021), Schreiner et al. (2022), and Heydemann et al. (2023) [36,37,54,55,56]. After anesthesia, animals were inoculated with either phosphate-buffered saline (PBS, mock infection) or infected with 10^5^ plaque-forming units (PFUs) of SARS-CoV-2. Animals were sacrificed at 1, 3, and 6 dpi, and lung samples were collected in 10% formalin during subsequent necropsies. Mock-infected control animals were selected randomly from all three time points, regardless of sex.

Formalin-fixed lung samples of both studies were routinely processed, including embedding in paraffin and trimming of 2–4 µm thick serial sections. Subsequently, sections were mounted on SuperFrost^®^Plus slides (Glasbearbeitungswerke GmbH & Co. KG, Braunschweig, Germany).

### 4.2. Viral RNA Levels and Viral Titers

The data of viral titers and viral mRNA levels of selected animals were published previously in Armando, Beythien [35] for experiment 1 and Stanelle-Bertram, Beck [36] for experiment 2 and were taken from the original raw datasets.

For experiment 1, viral RNA was extracted from lung tissue samples using a QIAmp Viral extraction kit in accordance with the manufacturer’s instructions, and qRT-PCR assays were performed. Virus infectious titers were determined in Vero cells for SARS-CoV-2 614G_Bavpat-1_ and the VOCs Gamma and Delta. The VOC Omicron was propagated and titrated in Calu-3 cells due to its limited replication in VeroE6 cells [35].

For experiment 2, viral RNA was isolated from homogenized lung samples using the QIAamp Viral RNA Mini Kit (QIAGEN GmbH, Hilden, Germany) in accordance with the manufacturer’s instructions. Subsequently, RNA levels were determined by qRT-PCR using the RealStar SARS-CoV-2 RT-PCR Kit RUO (Altona Diagnostics GmbH, Hamburg, Germany). For the assessment of viral titers, lung tissue homogenates and cell culture supernatants were titrated for SARS-CoV-2 on VeroE6 cells and the plaques were visualized by crystal violet staining [36].

### 4.3. Immunofluorescence

The antibodies and dilutions used for the immunolabeling and visualization of SARS-CoV-2 antigens and dsRNA intermediates are detailed in Table 1.

Immunofluorescent co-detection of SARS-CoV-2 S and NP was performed using the Vector^®^ TrueVIEW^®^ Autofluorescence Quenching Kit (Vector Laboratories, Inc., Newark, CA, USA). Tissue sections were deparaffinized and rehydrated, and heat-induced antigen retrieval was performed in citrate-Na_2_H_2_EDTA for 20 min in a microwave at 800 W. Unspecific binding reactions were reduced by subsequent blocking using normal goat serum (diluted 1:5 in PBS). Primary antibodies were incubated overnight at 4 °C. For negative controls, the primary antibodies were replaced with rabbit serum (diluted 1:3000 in PBS) or BALB/c mouse ascitic fluid (diluted 1:100 in PBS). Secondary antibodies were incubated for 60 min at room temperature (RT) in the dark. Sections were washed three times with PBS after each step. Autofluorescence quenching was performed for 5 min at RT in the dark. Finally, stained sections were washed with deionized water and mounted using Vectashield^®^HardSet anti-fade mounting medium containing 4′,6-diamidino-2-phenylindole (DAPI) for nuclear counterstaining (Vector Laboratories, Inc., Newark, CA, USA).

For the co-detection of SARS-CoV-2 S and dsRNA intermediates, a variation in the described protocol using an Alexa Fluor™ 488 Tyramid SuperBoost Kit (Invitrogen by Thermo Fisher Scientific GmbH, Vienna, Austria) was employed to enhance dsRNA immunolabeling. In addition to the aforementioned steps, endogenous peroxidase activity was blocked after the rehydration of sections by submersion in 85% ethanol solution containing 3% H_2_O_2_ for 30 min at RT. Unspecific antibody binding was blocked using the Alexa Fluor™ 488 Tyramid SuperBoost Kit blocking component. Tyramidin signal boosting was performed for 4 min at RT following secondary antibody application.

### 4.4. 2-Plex Fluorescent In Situ Hybridization (FISH)

FISH 2-plex assay was performed using the ViewRNA^TM^ ISH Tissue Core Kit (Invitrogen by Thermo Fisher Scientific GmbH, Vienna, Austria) and the ViewRNA^TM^ ISH Tissue Blue Module (Invitrogen by Thermo Fisher Scientific GmbH, Vienna, Austria). Two differently labeled probes were designed for the detection of genomic RNA of SARS-CoV-2 S (ViewRNA^TM^ Type 1 probe set, signal detection by ViewRNA^TM^ ISH Tissue Core Kit using Fast Red) and a second probe binding to the negative-sense RNA of the SARS-CoV-2 NP (ViewRNA^TM^ Type 6 probe set, signal detection by ViewRNA^TM^ ISH Tissue Blue Module using Fast Blue) similarly to Liu et al. (2020) [6]. The ViewRNA^TM^ probe sets were produced by Thermo Fisher Scientific (Invitrogen by Thermo Fisher Scientific GmbH, Vienna, Austria) according to the selected sequences (Supplementary Table S2). FISH was performed in accordance with the manufacturer’s protocol. One day prior to hybridization, the sections were baked in an oven at 60 °C for one hour. After deparaffinization, the sections were air-dried for 5 min at RT. Subsequent to heat pretreatment at 85–90 °C for 20 min, protease digestion was performed at 40 °C for 10 min. Afterwards, the sections were incubated with probe sets at 40 °C for 2 h. For negative controls, the sections were incubated only with Probe Set Diluent. Signal amplification and signal detection were achieved by incubation with Premplifier, Amplifier, alkaline phosphatase (AP)-linked Label Probe, AP Enhancer, and the subsequent application of Fast Blue and Fast Red substrates. The sections were counterstained with Meyer’s hemalaun and mounted with aqueous mounting medium (Dako Fluorescence Mounting Medium, Dako North America Inc., Carpinteria, CA, USA).

### 4.5. Digital Image Analysis

The stained tissue sections were digitalized using an Olympus VS200 Slide scanner. Subsequent digital image analysis was performed using the open source software package QuPath version 0.5.1 for digital pathology image analysis [57]. For experiment 1, one longitudinal and one transversal cross section of the left lung lobe were analyzed. For experiment 2, up to four cross sections of lung tissue from different lobes were investigated. Lung tissue was detected as region of interest (ROI) using pixel classifiers based on staining-specific thresholds of the DAPI channel and the size exclusion of objects smaller than 10^6^ µm^2^. Staining artifacts, including tissue folds, were removed from ROIs using channel-specific thresholds and size exclusion parameters as well as manual annotations. Individual cells were identified using the QuPath feature “cell detection”. Within this feature, individual cells were identified based on a DAPI channel threshold. To determine the amount of immunolabeled cells, stain- and channel-specific sets of artificial neural network machine learning algorithm-based object classifiers were used. Training included the annotation of ≥150 immunopositive as well as -negative cells per image.

### 4.6. Statistical Analysis

The total amounts of detected and immunolabeled cells per animal and staining as well as the percentages of immunolabeled cell populations were calculated using Microsoft Excel version 2408 (Build 17928.20114; Microsoft Corp., Redmond, WA, USA) for Windows. To correct for false detections, the maximum percentage of detected immunolabeled cells within the PBS group of experiment 2 was subtracted from all other detections as a staining-specific correction factor. Statistical analysis and graph design were performed using GraphPad Prism 10.3.0 (GraphPad Software, Inc., San Diego, CA, USA) for Windows™. Data were tested for normality using the Shapiro–Wilk test. Significant differences were investigated using Kruskal–Wallis tests, followed by pairwise comparisons obtained by two-tailed Mann–Whitney U tests. Correction for multiple comparisons was performed using the Benjamini–Hochberg correction. Statistical significance was accepted at exact *p*-values of ≤0.05. Correlation of virological parameters and immunolabeling results was calculated as two-tailed Spearman’s rank correlation coefficient.

## 5. Conclusions

The detection of dsRNA intermediates of viral replication with commercially available anti-dsRNA antibodies represents a suitable alternative to viral antigen detection during the early phase of SARS-CoV-2 infections. However, available antibodies possess significant differences in detection efficacy. The evaluation of cell portions double-labeled for viral antigen and dsRNA led to the efficient assessment of differences in viral replication activity within different variants of SARS-CoV-2 and showed a strong positive correlation with viral titers. Anti-dsRNA antibodies are therefore an interesting tool for the histopathological evaluation of in vivo studies with restricted time points or reduced availability of virological samples.

## Figures and Tables

**Table 1:** Primary antibodies, supplier, dilution, clonality, and host species as well as secondary antibodies used for immunofluorescent investigations.

Primary Antibody	Supplier (Catalog Number)	Dilution	Clonality, Host Species	Secondary Antibody (Supplier; Dilution)
SARS-CoV NP	Sino Biological, Peking, China (40143-MM05)	1:16,000	Monoclonal, mouse	Goat anti-Mouse Cy2 (Dianova, Hamburg, Germany, 1:200)
SARS-CoV-2 S2	Sino Biological, Peking, China (40590-T62)	1:4000	Polyclonal, rabbit	Goat anti-rabbit Cy3 (Dianova, Hamburg, Germany, 1:200)
dsRNA J2	Jena Bioscience, Jena, Germany (RNT-SCI-10010200)	1:300	Monoclonal, mouse	Alexa Fluor™ 488 Tyramid SuperBoost Kit (Invitrogen by Thermo Fisher Scientific, Vienna, Austria, 1:200)
dsRNA K1	Jena Bioscience, Jena, Germany (RNT-SCI-10020200)	1:150	Monoclonal, mouse	Alexa Fluor™ 488 Tyramid SuperBoost Kit (Invitrogen by Thermo Fisher Scientific, Vienna, Austria, 1:200)
dsRNA 9D5	Absolute antibody, Wilton, UK (Ab00458-1.1)	1:100	Monoclonal, mouse	Alexa Fluor™ 488 Tyramid SuperBoost Kit (Invitrogen by Thermo Fisher Scientific, Vienna, Austria, 1:200)

dsRNA, double-stranded RNA; SARS-CoV NP, severe acute respiratory syndrome coronavirus nucleoprotein; SARS-CoV-2 S2, severe acute respiratory syndrome coronavirus 2 spike protein.

## Data Availability

The original data presented in the study are provided in Supplementary Table S1. Further information is available on reasonable request from the corresponding author.

## Supplementary Materials

The following supporting information can be downloaded at: https://www.mdpi.com/article/10.3390/ijms252111425/s1.
