# Supplementary material for: Detection of Double-Stranded RNA Intermediates During SARS-CoV-2 Infections of Syrian Golden Hamsters with Monoclonal Antibodies and Its Implications for Histopathological Evaluation of In Vivo Studies
Source: Int J Mol Sci. 2024 Oct 24;25(21):11425. doi: 10.3390/ijms252111425 (PMC11546166; doi:10.3390/ijms252111425)
Supplement: Supplementary file 1 [file ijms-25-11425-s001.zip › DsRNA-JMS_Supplementary_Figures_100924.pdf]

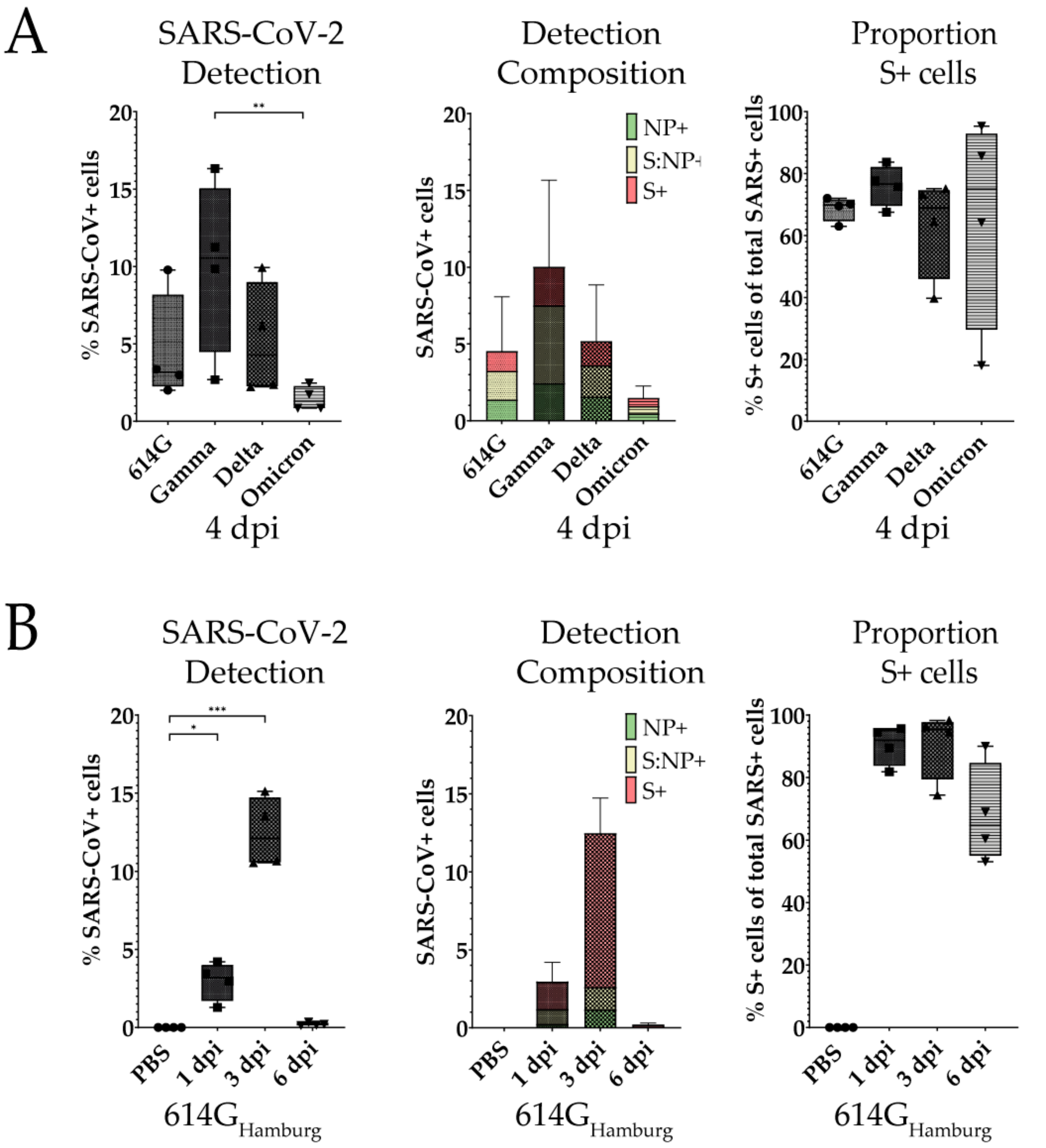

Supplementary Figure S1. Comparison of SARS-CoV-2 spike protein (S) and nucleoprotein (NP) detection in SARS-CoV-2 infected hamsters. (**A**) Detection of SARS-CoV-2 S and NP at 4 days post infection (dpi) in $614G_{Bavpat-1}$ and variants of concern (VOCs) Gamma, Delta, and Omicron infected animals. (**B**) SARS-CoV-2 S and NP detection at 1, 3 and 6 dpi with early strain $614G_{Hamburg}$. Data are shown as box and whisker plots. The bounds of the box plot indicate the 25th and 75th percentiles, the bar indicates medians, and the whiskers indicate minima and maxima. Composition of detect cell populations are shown stacked bars of mean proportion of immunolabeled cell populations. Whiskers indicated maximum of total detected cells within group. Data were tested by two-tailed Mann–Whitney-U tests followed by Benjamini–Hochberg correction. A p-value of ≤0.05 was chosen as the cut-off for statistical significance. N = 4 animals/group. Exact p values are represented as * with * p<0,05, ** p<0,01, and *** p<0,001.

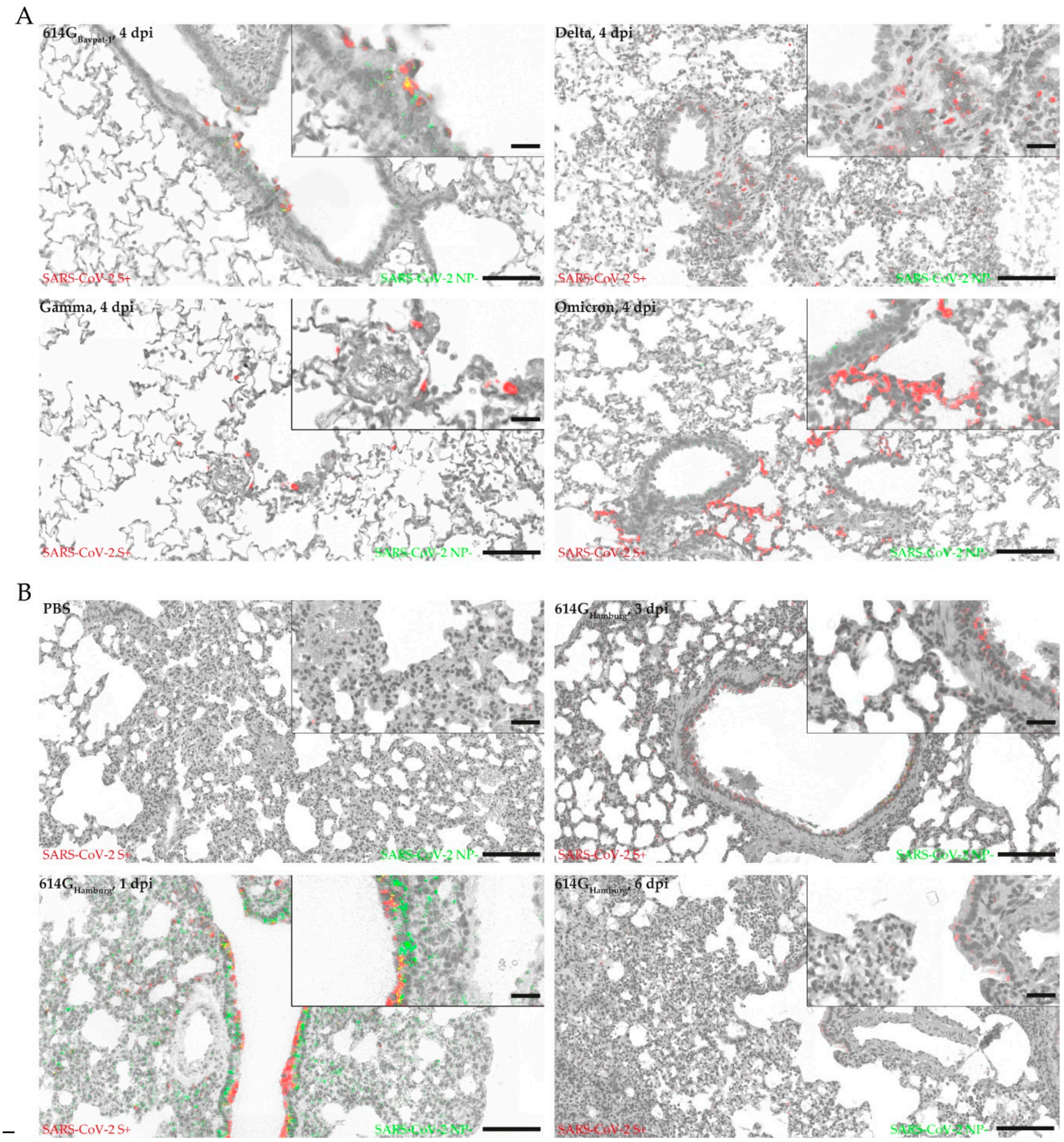

Supplementary Figure S2. Representative images of fluorescent 2-plex in situ hybridization of positive sensed SARS-CoV-2 spike protein (S) coding RNAs (SARS-CoV-2 S+) and negative sensed SARS-CoV-2 nucleoprotein (NP) coding RNA intermediates (SARS-CoV-2 NP-). (**A**) Detection of SARS-CoV-2 RNA subspecies at 4 days post infection (dpi) in $614G_{Bavpat-1}$ and variants of concern (VOCs) Gamma, Delta, and Omicron infected animals. (**B**) Detection of SARS-CoV-2 RNA subspecies at 1, 3 and 6 dpi with early strain $614G_{Hamburg}$. Data are shown as box and whisker plots. Bars represent 100 µm and 25 µm (inlets) respectively.

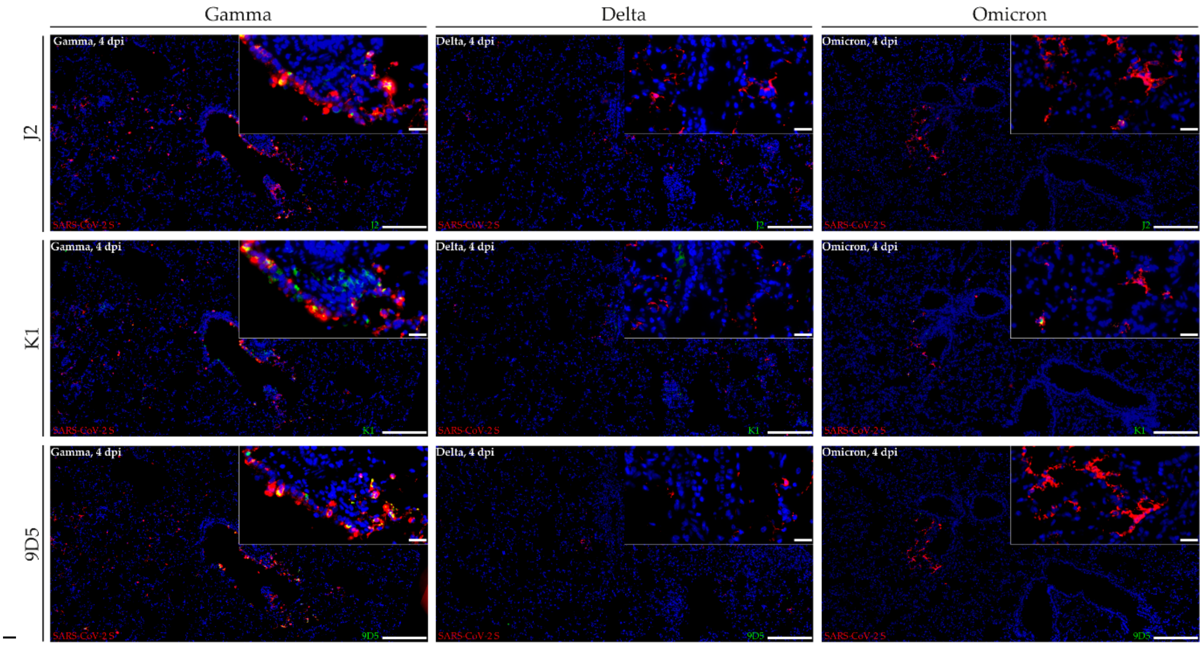

Supplementary Figure S3. Representative images of double-stranded RNA (dsRNA) and SARS-CoV-2 spike protein (S) co-detection in an animal infected with 614G$_{\text{Bavpat-1}}$ and variants of concern (VOCs) Gamma, Delta, and Omicron at 4 days post infection. Bars indicate 200 µm and 20 µm (insert) respectively.

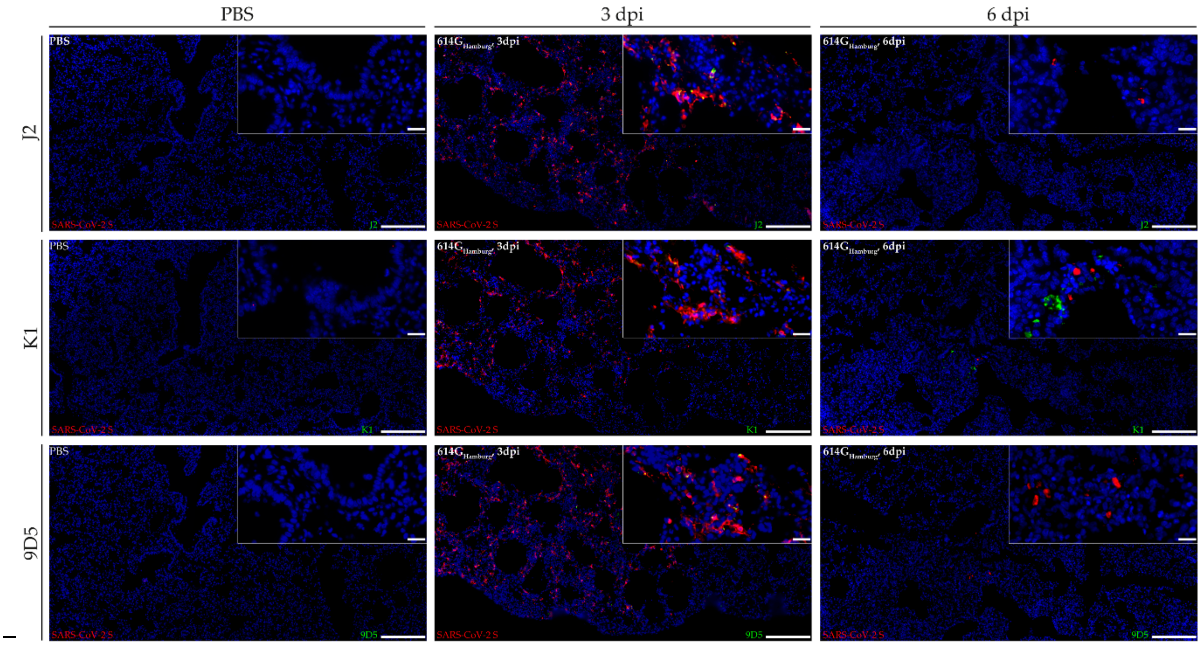

Supplementary Figure S4. Representative images of double-stranded RNA (dsRNA) and SARS-CoV-2 spike protein (S) co-detection in animal infected with 614G$_{\text{Hamburg}}$ at 3 and 6 days post infection or mock infection with phosphate-buffered saline (PBS). Bars indicate 200 µm and 20 µm (insert) respectively.
