# Supplementary material for: Detection of Double-Stranded RNA Intermediates During SARS-CoV-2 Infections of Syrian Golden Hamsters with Monoclonal Antibodies and Its Implications for Histopathological Evaluation of In Vivo Studies
Source: Int J Mol Sci. 2024 Oct 24;25(21):11425. doi: 10.3390/ijms252111425 (PMC11546166; doi:10.3390/ijms252111425)
Supplement: Supplementary file 1 [file ijms-25-11425-s001.zip › Supplementary Table S2.pdf]

Supplementary Table S2. Nucleotide sequences chosen for probe design.

Target structure	Nucleotide sequence
Genomic RNA of spike protein	3′GUAAUGGUGUUGAAGGUUUUAAUUGUUACUUUCCUUUACAAUCAUAUGGUUUCCAACCC<br>ACUAAUGGUGUUGGUUACCAACCAUACAGAGUAGUAGUACUUUCUUUUGAACUUCUACAU<br>GCACCAGCAACUGUUUGUGGACCUAAAAAGUCUACUAAUUUGGUUAAAAACAAAUGUGUC<br>AAUUUCAACUUCAAUGGUUUAACAGGCACAGGUGUUCUUACUGAGUCUAACAAAAAGUUU<br>CUGCCUUUCCAACAAUUUGGCAGAGACAUUGCUGACACUACUGAUGCUGUCCGUGAUCCAC<br>AGACACUUGAGAUUCUUGACAUUACACCAUGUUCUUUUGGUGGUGUCAGUGUUAUAACAC<br>CAGGAACAAAUACUUCUAACCAGGUUGCUGUUCUUUAUCAGGAUGUUAACUGCACAGAAG<br>UCCCUGUUGCUAUUCAUGCAGAUCAACUUACUCCUACUUGGCGUGUUUAUUCUACAGGUU<br>CUAAUGUUUUUCAAACACGUGCAGGCUGUUUAAUAGGGGCUGAACAUGUCAACAACUCAU<br>AUGAGUGUGACAUACCCAUUGGUGCAGGUAUAUGCGCUAGUUAUCAGACUCAGACUAAUU<br>CUCCUCGGCGGGCACGUAGUGUAGCUAGUCAAUCCAUCAUUGCCUACACUAUGUCACUUGG<br>UGCAGAAAAUUCAGUUGCUUACUCUAAUAACUCUAUUGCCAUACCCACAAAUUUUACUAU<br>UAGUGUUACCACAGAAAUUCUACCAGUGUCUAUGACCAAGACAUCAGUAGAUUGUACAAU<br>GUACAUUUGUGGUGAUUCAACUGAAUGCAGCAAUCUUUUGUUGCAAUAUGGCAGUUUUUG<br>UACACAAUUAAACCGUGCUUUAACUGGAAUAGCUGUUGAACAAGACAAAAACACCCAAGA<br>AGUUUUUGCACAAGUCAAACAAAUUUACAAAACACCACCAAUUAAAGAUUUUGGUGGUUU<br>UAAUUUUUCACAAAUAUUACCAGAUCCAUCAAAACCAAG5′
Anti-genomic RNA of nucleoprotein	5′TTUGGCCTGUGTTGUGTCUGCUCTGCTCUTGGUTTGTTGCUUTTGTTTGGUGUUUTCUTCCUU<br>UTCTGCUGCUGGUUGUUGUGTCUCUGTTTGCTGTTTCTTCTGTCTCTGCGGTUUGGCTTGUGTTT<br>CUTCUGCCTTCTTCTTTTTGTCCTTTTTUGGCTCTGTTGGTGGGUUTGTTTTGTUTGCGTCUUTUTG<br>CTTUTTCUGCUUUUTGUCTTGUTCTTTGUUUTTTGGUTCTTTGTCUTCCUUTTTGUTGGCUCCTGT<br>GTUGGTCUUCCUCGTTCCCGUUGGTGTGUCTTCCUTGCCUUTGCGCGUCUTTCCGUUGUUCGC<br>TGUUGCGCTGGGGGCUUUTTGTGCUUTTTGCGGCCUUTGTTTGTUUTCUGTTCCTTGTCTGUTTU<br>GTTCCTGGTCCCCUUUUTTTCCTTGGGTTTGTTCTGGUCCUCGTCTGCCGUUUGCTTGTGTTUCU<br>TTGTUTGCTTTUGTGGCUGTUCGTTTTTGCCGUGGCTTCTTUGUUGCCTCUGCUGCUGUTTTCTT<br>UGTGUCUGTTTGGCCTTGTTGTTGTTGGCCTTTUCCUGUCUTTTTGCTCTCUUGCTGGTTCUUTCT<br>GTCUUGCUGCUGCUUUGCUUGUGCUGCUTCUCCGCCUTTGCCUGCCUTTCTUGCUGGUGUUG<br>TTCCCCTUCTGCTGCCTGGUGTTGUUTTTCTTGUUCTGTTGCGUCTUCGTGUTGUGGUUCGUGU<br>UGUGGCTTGUCTGCCGCCTCTGCTCCCTTCTGCGTUGUUGCCTTTTGGCUUTGTTGTTCCTTGUG<br>GUUGTTGTUGCUCGUTTGCUGCUTTGTTUGCUGGUTTGCGGGTGCCUUTGTGUTCTTTTGGTGT<br>UTTCUUGGCTCCCTCUGTTGCUUCCCUTUTGUTGCCGTCTTTGTTUGCUCCUTUGGGUUGTCCU<br>GCTTCTGGCCCUGTTCCTUGGTUGTUGUUUTUCCUTCTTGGUCTGUGUTCTTTCUTTTTUCCGTC<br>UCCUCCUCGUUTTCGTCTGGTUGCTCTTCGGTUGTUGCCUUTTTGGTCUTCTGGUCTGCTUTTGG<br>TGTTUUTTGGUUCGCCTTGTCCTCGUGGGUUTTTUUGGTCTTCCTTGCCUTGTTGUGTGUGUGCG<br>GTGUUCCUUGUCGCUGTUTTUTTGGGTUUUCCTTGGGGCCGUCGTTGTTTTGUTCGCGCCCCUC<br>TGCGTTCTCCUTTCTGGTTUCTGCCUGTTGUUTCTGUGGGTCCUCCUUUCGTUUTGCGGGGTGC<br>UTTTCGCTGUTTTTGGGGTCCUTTUTCUGUCUT3′
